# Development of Dose‐Response Models for the Ingestion Exposure Route and Stillbirth Outcome for *Listeria monocytogenes*

**DOI:** 10.1111/risa.70204

**Published:** 2026-03-17

**Authors:** Tyler Stump, Carly Gomez, Jade Mitchell

**Affiliations:** ^1^ Department of Engineering Education The Ohio State University Ohio, Columbus USA; ^2^ Department of Biosystems and Agricultural Engineering Michigan State University East Lansing Michigan USA

**Keywords:** dose‐response, *Listeria monocytogenes*, sensitive population, stillbirth

## Abstract

Foodborne listeriosis can cause stillbirth in pregnant individuals, prompting numerous population‐specific food safety guidelines. Dose‐response models are employed in quantitative microbial risk assessments (QMRAs) to develop such guidelines; however, pregnant hosts are under‐considered when developing dose‐response models, and the few published dose‐response models for pregnant hosts do not consider the biological plausibility of the model. Therefore, this study aims to develop biologically plausible dose‐response models for pregnant hosts using infection and stillbirth as health outcomes. The inclusion criteria of dose‐response data for this analysis required reporting of (i) quantified dose, (ii) *Listeria monocytogenes* as the target pathogen, (iii) infection and/or stillbirth as endpoints, and (iv) specificity to pregnant hosts. The datasets were fit individually and as pooled sets to exponential and beta‐Poisson dose‐response models using Maximum Likelihood Estimation (MLE). To establish uncertainty around the MLE estimates, 10,000 bootstrapped iterations were fit to the models. Recommended dose‐response models for endpoints of stillbirth and infection were generated. The recommended model for infection was based on pooled guinea pig and gerbil maternal infection data fit to the beta‐Poisson model, with parameters of: *α* = 0.0843 and *N_50_* = 1.01 × 10^8^. The recommended stillbirth dose‐response model utilized a pooled guinea pig fetal brain infection and stillbirth dataset fit to the beta‐Poisson model parameters of *α* = 0.575 and *N_50_* = 1.31 × 10^7^. Integrating such models into QMRAs for pregnant hosts supports a more health‐protective assessment and decision‐making process by separating sensitive populations from the general public when possible.

## 1 Introduction

*Listeria monocytogenes* remains a pathogen of concern within food handling environments and food systems. The facultative anaerobic bacterium can grow in challenging conditions such as refrigeration temperatures of 4°C (Ryser and Marth 1999), and *L. monocytogenes* biofilms can survive and develop resistance to peroxidase and chlorine sanitizers typically used in food processing environments (Pan et al. 2006). Moreover, the pathogen can persist up to 8 years in processing environments and is frequently isolated from consumer homes and retail settings (Ferreira et al. 2014). *L. monocytogenes* can cause adverse human health effects such as infection, stillbirth, and/or death (Ryser and Marth 1999). This creates public health concerns, particularly for pregnant individuals exposed to high‐risk foods.

Environmental monitoring programs (Simmons and Wiedmann 2018), consumer education strategies (Centers for Disease Control and Prevention 2023), and most notably, the US Food and Drug Administration's “zero‐tolerance” policy for *L. monocytogenes* in ready‐to‐eat foods (U.S. Food and Drug Administration 2020) exist as the basis for *L. monocytogenes* risk management. Despite these efforts, approximately 1250 Americans develop listeriosis each year, with estimated hospitalization and death rates of 86% and 14%, respectively (Scallan Walter et al. 2025). In addition, listeriosis cases in the European Union (EU) and the European Economic Area (EEA) continue to rise (European Centre for Disease Prevention and Control 2024). As such, *L. monocytogenes* causes the third‐most deaths of all bacterial foodborne pathogens (Scallan Walter et al. 2025). Vulnerable populations such as pregnant individuals face even higher rates of morbidity and mortality, with particular concern for fetal infection, which causes stillbirth in 25% of cases (Segado‐Arenas et al. 2018). Pregnancy‐associated listeriosis cases account for 14% of all listeriosis cases, but some outbreaks predominantly impact pregnant individuals, such as the Mexican‐style cheese outbreak in 2000 (MacDonald et al. 2005). Several recent listeriosis outbreaks sickened numerous pregnant people and resulted in stillbirth, the 2022 ice cream outbreak (1 stillbirth) (Centers for Disease Control and Prevention 2022), the 2021 queso fresco outbreak (2 stillbirths) (Centers for Disease Control and Prevention 2021), and the 2020 enoki mushroom outbreak (2 stillbirths) (Centers for Disease Control and Prevention 2020).

Dose‐response models characterize the probability of a specific endpoint given a particular pathogen dose. Crucially, observable maternal listeriosis does not necessarily indicate or predict fetal infection and stillbirth (Wang et al. 2021). Many pregnant individuals are asymptomatic or have generalized symptoms, such as headache, fever, and diarrhea, but *L. monocytogenes* can still cause fetal death by crossing through the placenta, reducing fetal tolerance to Treg cells, or by vertical transmission (Wang et al. 2021). Thus, it is vital to distinguish stillbirth as an endpoint for listeriosis in pregnancy. Dose‐response relationships are grounded in the context of the adverse health endpoint, the pathogen, the population of interest, and the route of exposure, with public health risk management favoring the most representative model (U.S. Food and Drug Administration, 2003).

Model biological plausibility is essential when developing mechanistically representative dose‐response models for microbial pathogens (Haas et al. 2014). In short, biologically plausible dose‐response models must allow for actual doses at low‐level exposures. For instance, an average concentration below 1 CFU per volume should not imply that hosts consume fractions of organisms; instead, many hosts will consume 0 CFU, and a small portion will consume 1 or more CFU. Second, a biologically plausible dose‐response model must also consider the host‐pathogen relationship that may allow microbes to survive and reproduce within a susceptible host (Haas et al. 2014). Given these criteria, and the derivations provided by Haas et al., there are two biologically plausible dose‐response models typically used in microbial dose‐response modeling the exponential and the beta‐Poisson, though other model forms may be developed (Haas et al. 2014).

Dose‐response models have been published to support *L. monocytogenes* risk assessments for over two decades. An early dose‐response model utilized the exponential model to describe “susceptible populations” for infection as an outcome (Buchanan et al. 1997). The authors targeted susceptible populations by utilizing general population surveillance data and assuming those represented within the data were immunocompromised or susceptible populations, and Lindqvist and Weslöö (Lindqvist and Westöö 2000) applied Buchanan's exponential dose‐response parameter in their assessment for a “high‐risk” group. However, the representation of a generally susceptible or high‐risk population does not account for complex mechanistic differences between infection and endpoints in pregnant hosts and in immunocompetent individuals. These approaches can result in risk assessments that are not appropriate for pregnant individuals (U.S. Food and Drug Administration 2003). Lindqvist and Westöö (2000) and Farber et al. (1996) also employed empirical data to generate dose‐response curves, leveraging either only the Weibull‐Gamma (WG) model or both the WG model and the exponential dose‐response model; however, these analyses did not consider both biologically plausible dose‐response models (Farber et al. 1996; Lindqvist and Westöö 2000). Ideally, both models would have been tested to determine the best‐fitting biologically plausible model.

A few listeriosis dose response models specific to pregnant individuals are available in the literature. A recent listeriosis dose‐response model attempted to account for differences in host susceptibility and strain virulence for sensitive population subgroups, including pregnant women, using relative risks (Pouillot et al. 2015). While this is a positive step towards accounting for vulnerable populations, it is unclear if linear transformations such as relative risks can accurately be applied across pathologically complex conditions, and the endpoint of infection is not equivalent to stillbirth. In addition, Roulo et al. conducted a feeding study in gerbils, but because doses less than 10^9^ failed to produce stillbirths, the logistic dose‐response model was based on fetal tissue invasion, which does not necessarily correspond to stillbirth, and beta‐Poisson and exponential models were not tested (Roulo et al. 2014). However, the fetal tissue invasion data may be useful for pooling with dose‐response data with similar endpoints.

On the other hand, Williams et al. and Smith et al. compared stillbirths in pregnant guinea pigs and rhesus monkeys, respectively, following oral exposure to *L. monocytogenes* (Smith et al. 2008; Williams et al. 2007). Rhesus monkeys have an analogous placenta and reproductive cycle to humans, and equivalent listeriosis stillbirth pathogenesis (Smith et al. 2003), while guinea pigs have an identical E‐cadherin sequence to humans (Williams et al. 2007). Williams et al. and Smith et al. fit their experimental dose‐response data to exponential and log‐logistic models, but not the beta‐Poisson model. Because the data from these studies were not fit to both biologically plausible dose‐response models, refitting the original and pooled data with biologically plausible models could allow for the development of a more comprehensive, feeding study‐driven dose‐response model for listeriosis stillbirth. This demonstrates a need for biologically plausible dose‐response models specific to oral exposures of *L. monocytogenes* in pregnant hosts, to support more representative risk assessments.

Consequently, the goal of this study was to compare dose‐response model performance and endpoint differences for previously published, newly fit, and pooled listeriosis stillbirth dose‐response data. There are currently very few dose‐response models available for constructing representative risk assessments for sensitive populations. This dose‐response model will allow for the development of improved risk management strategies for pregnant individuals—a sensitive population.

## 2 Methods

### 2.1 Dose‐Response Assessment

To identify datasets relevant to the target goal, four criteria were considered: (i) dose was quantified, (ii) *L. monocytogenes* was the hazardous pathogen, (iii) the number of hosts reaching each endpoint was specified, and (iv) the experiments were conducted with the target population (pregnant animals).

#### 2.1.1 Infection Model Dose‐Response Data

The literature available provided two applicable datasets, Golnazarian et al. and Williams et al. (Golnazarian et al. 1989; Williams et al. 2007). Smith et al. was not applicable to the infection data, as the tested endpoint was stillbirth (Smith et al. 2008). Golnazarian et al. monitored infection (ID50) in pregnant mice (12–14‐day gestation) orally exposed to *L. monocytogenes* F5817 (Golnazarian et al. 1989). The second study included was Williams et al.’s multi‐endpoint dose‐response analysis on pregnant guinea pigs following oral ingestion of 10^4^–10^8^ CFU *L. monocytogenes* on gestation Day 35 (Williams et al. 2007). The number of dams with infected fetuses was recorded and utilized in the present study's infection modeling. The third animal model considered for inclusion in this study was identified in Roulo et al. 2014. Roulo et al. utilized used gerbil animal models to understand the dose‐response relationship to *L. monocytogenes* by exposing the gerbils to 3‐, 5‐, 7‐, and 9‐log levels of oral exposure. The data used herein for the infection endpoint in pregnant hosts are summarized in Table 1.

**TABLE 1:** Dose‐response data for pregnant hosts exposed orally to *L. monocytogenes*.

Animal model	Endpoint	Dose (CFU)	Positive responses	Negative responses	*L. monocytogenes* strain (serotype)	Reference
Mouse	INF	32.36	0	2	F5817 (4b)	(Golnazarian et al. 1989)
		346.74	1	1		
		549.54	2	0		
		4265.81	2	0		
Guinea pig	INF	10,000	0	4	12443 (1/2a)	(Williams et al. 2007)
		100,000	2	11		
		1,000,000	2	9		
		10,000,000	3	9		
		100,000,000	3	4		
Gerbils	INF	1000	0	4	12443 (1/2a)	(Roulo et al. 2014)
		100,000	1	3		
		10,000,000	2	2		
		1,000,000,000	4	0		

#### 2.1.2 Stillbirth Model Dose‐Response Data

Three datasets confirming stillbirth, or related endpoint, aligned with all selection criteria described above. Williams et al.’s guinea pig study provided two of such datasets—Guinea Pig‐Fetal Brain (GP‐FB) and Guinea Pig‐Stillbirth (GP‐STILL) (Williams et al. 2007). The endpoint in the GP‐FB dataset was confirmation of *L. monocytogenes* in fetal brain tissue. This endpoint occurred in every stillbirth, and never in non‐stillbirth cases, demonstrating that when the fetal brain was infected, there was a high likelihood that stillbirth would occur (Williams et al. 2007). Thus, the dataset was considered distinct for the development of a stillbirth dose‐response model. The same authors also documented the dose‐response endpoint of fetal mortality by observing fetal tissues to determine fetal viability (Williams et al. 2007, Table 5). Exposure doses and positive/negative responses were indicated for both endpoints. The third dataset was from Smith et al.’s study, in which pregnant rhesus monkeys were orally exposed to 10^2^–10^10^ CFU of *L. monocytogenes* and stillbirths were recorded (Smith et al. 2008). The three datasets utilized herein for stillbirth dose‐response model development are summarized in Table 2.

**TABLE 2:** Stillbirth dose‐response data for pregnant animals orally exposed to *L. monocytogenes*.

Animal model	Endpoint	Dose (CFU)	Positive responses	Negative responses	*L. monocytogenes* strain (serotype)	Reference
Guinea Pig (fetal brain)	Stillbirth	10,000	0	18	12443 (1/2a)	(Williams et al. 2007)
		100,000	0	62		
		1,000,000	5	36		
		10,000,000	11	20		
		100,000,000	12	5		
Guinea pig	Stillbirth	10,000	0	18	12443 (1/2a)	(Williams et al. 2007)
		100,000	0	64		
		1,000,000	6	35		
		10,000,000	18	24		
		100,000,000	20	1		
Rhesus monkey	Stillbirth	316.2	0	1	12443 (1/2a)	(Smith et al. 2008)
		1584.9	2	6		
		19,952.6	0	3		
		125,892.5	1	4		
		158,4893.2	2	4		
		12,589,254.1	2	3		
		125,892,541.2	2	2		
		39,810,717,055	1	0		

#### 2.1.3 Dose‐Response Model Fitting

Maximum likelihood estimation (MLE) methods were utilized as described by Haas et al. to optimize the dose‐response model parameters (Haas et al. 2014). Models were fit to dose‐response datasets with previously developed and published computer code in the statistical programming language, “R,” (R Core Team 2021; Weir et al. 2017). The two models considered were the exponential (Equation 1) and the approximate beta‐Poisson (Equation 2).

(1) $$P\left(d\right)=1-e^{-kd}$$

The exponential model is a single parameter model that connects the probability of a response, *P(d)*, to an exposure dose, *d*, with the parameter *k*, which represents the probability of any one organism surviving to initiate infection. The approximate beta‐Poisson model has two parameters, *α* and *N_50_*, where *α* represents the shape parameter in the beta distribution and *N_50_* is the dose affecting 50% of the population.

(2)

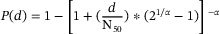




Goodness of fit and model comparison tests within the R code were utilized to determine the best‐fitting models for each data set. Model deviance *Y* (Equation 3) was compared to critical chi‐squared test values (*χ* ^2^), the 95% confidence value of the *χ* ^2^ distribution with degrees of freedom equivalent to the number of data points (dose groups) minus the number of parameters in the model. Models considered a good fit had a *Y* that was less than the corresponding critical *χ* ^2^ value. The model fits to the exponential and beta‐Poison were then compared by subtracting *Y_beta‐Poisson−_Y_exponential_*, and comparing the difference in model deviances, to a *χ* ^2^ distribution with 1degree of freedom. The more parsimonious model (the exponential model) was rejected if the difference in deviances was greater than the critical *χ* ^2^ value. This approach was used to identify a recommended model for individual dataset and each health endpoint. Model parameter confidence intervals were produced through bootstrapping and refitting the original data sets (Haas et al. 2014).

Individual datasets were also combined to conduct pooling analyses. Pooling analyses are done for two primary reasons: (1) when multiple strains of microorganisms or hosts exhibit similar dose‐response patterns, it strengthens the inference that another strain will behave similarly. Therefore, using a diverse set of animal models enhances the robustness of dose‐response analysis. Confidence in extrapolating to a new, unobserved species improves when data from multiple animal species are integrated. (2) A larger pool of data points reduces uncertainty, resulting in narrower confidence intervals around the dose‐response curves.

After models were fit to individual and combined datasets, the differences in minimized deviances, Δ, were used to compare the fits from the pooling analysis with the individual dataset fits in order to optimize a model for each endpoint according to Equation 4. The null hypothesis was rejected, and the datasets could be pooled, if the differences between the pooled model's deviance and the sum of individual models’ deviances was less than the critical *χ* ^2^ value with degrees of freedom equal to the total parameters from the combined individual models minus the parameters in the model fit to the combined data set (Haas et al. 2014).

(3) $$Y=-2\;\sum_{i=1}^{k}\left[P_{i}\ln\left(\frac{\pi_{i}}{\pi_{i}^{0}}\right)+\left(N_{i}\;-\;P_{i}\right)\ln\left(\frac{1\;-\;\pi_{i}}{1\;-\;\pi_{i}^{0}}\right)\right]\;$$

where *Y* is the deviance (or −2 times the log‐likelihood ratio); *P_i_* is the number of subjects with positive responses; *π_i_* is the model‐predicted response, ${\pi_{i}}^{o}$ is the response based on the observations in the study; and *N_i_* is the total number of subjects.

(4) $$\Delta \;=\left(Y_{pooled}-\sum_{i=1}^{n}Y_{i}\right)\;\;>\;\chi_{best\;fit,\;df}^{2}\;$$

where *Y_pooled_* is the deviance of the pooled model and *Y_i_* is the deviance of a model fit to an individual model. $\chi_{best\;fit,\;df}^{2}$ is the critical *χ* ^2^ value), the 95% confidence value of the *χ* ^2^ distribution with df, degrees of freedom equal to the total parameters from the combined individual models minus the parameters in the model fit to the combined data set.

## 3 Results

### 3.1 Model Fitting

#### 3.1.1 Infection Individual Datasets

**FIGURE 1:**
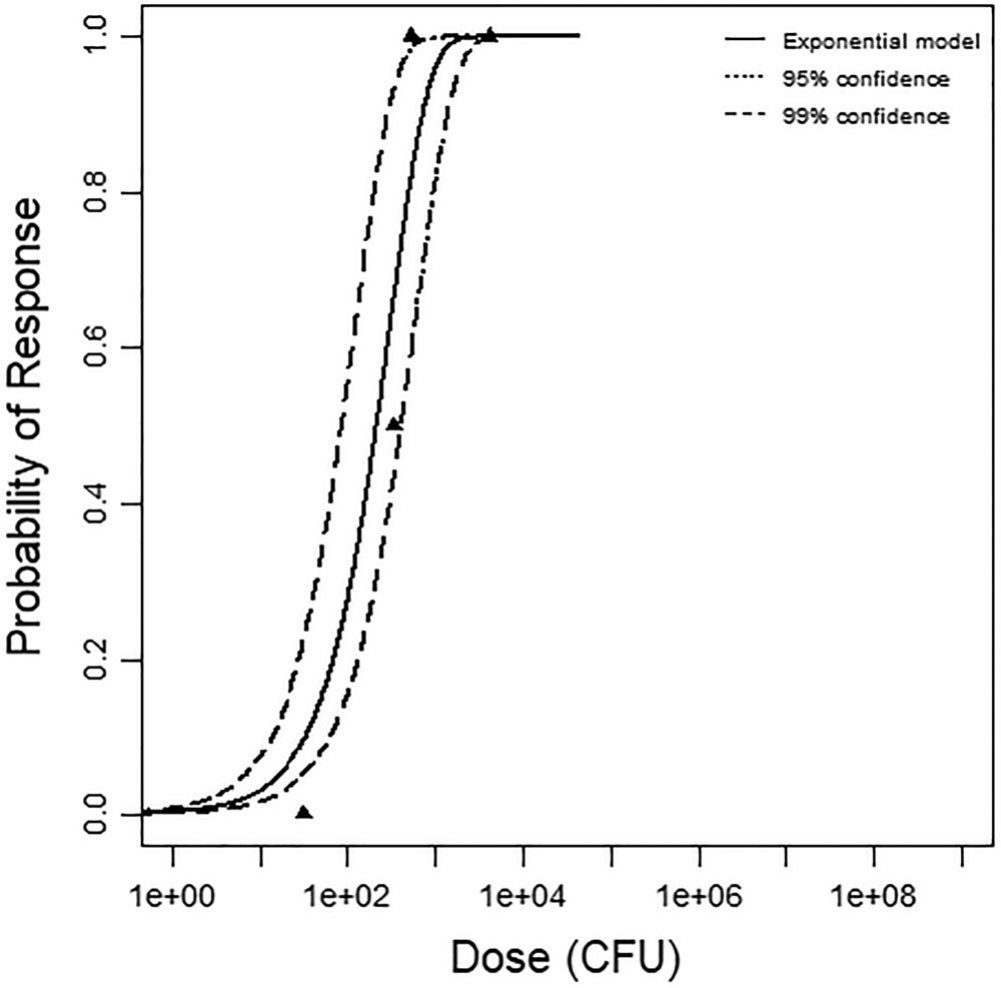
Best‐fitting model (exponential) for the mouse infection dataset, with 95% and 99% confidence bounds.

**FIGURE 2:**
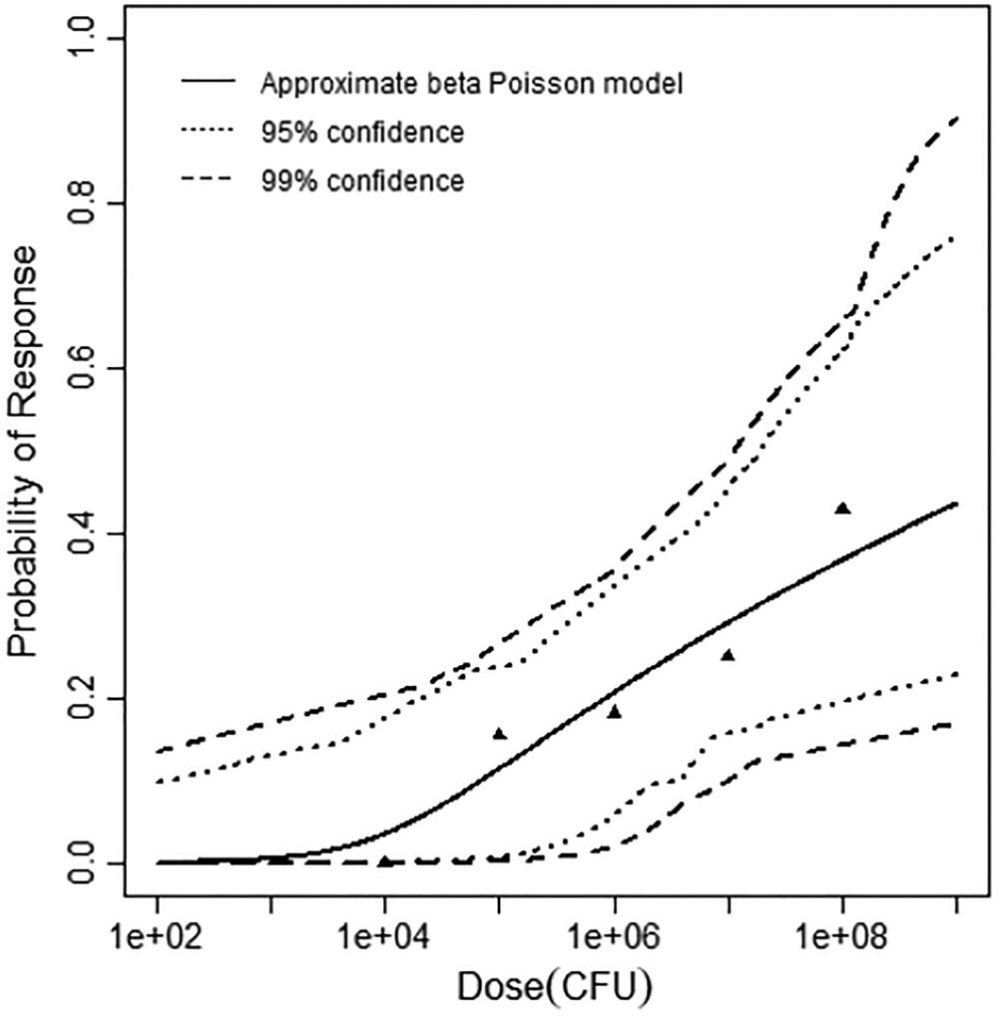
Best‐fitting model (beta‐Poisson) for the guinea pig infection dataset, with 95% and 99% confidence bands.

**FIGURE 3:**
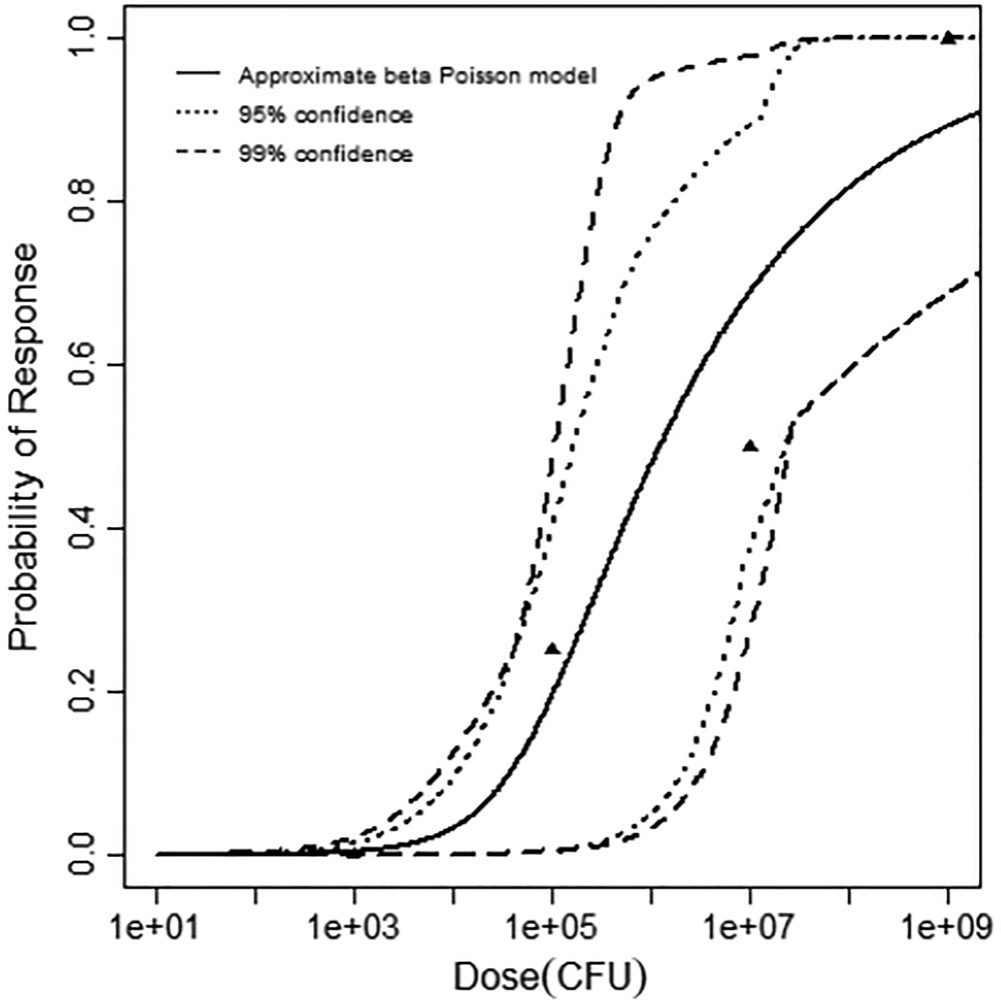
Best‐fitting model (beta‐Poisson) for the gerbil infection dataset, with 95% and 99% confidence bands.

For the Golnazarian et al. data set—MOUSE‐INF‐ (Golnazarian et al. 1989), the minimized deviances were 1.4166 and 1.4168 for the exponential and beta‐Poisson models, respectively (Table 3), indicating acceptable fit to both models. The best‐fitting model was the exponential model, as the difference in the deviances is less than the critical *χ* ^2^ value at 1 degree of freedom (3.842). Alternatively, for the guinea pig—infection model, only the beta‐Poisson model was a good fit, with minimized deviance of 0.7247. Both models were a good fit for the gerbil infection data (Roulo et al. 2014); however, the beta‐Poisson was selected as the best‐fitting model because the difference in the deviances of the two models (3.439) was less than the critical *χ* ^2^ value at 1 degree of freedom. All modeling results are outlined in Table 3, and graphs of the best‐fitting models for each dataset are shown in Figures 1, 2, 3.

**TABLE 3:** Infection individual dataset model fitting results.

Dataset	Analysis type	Dose‐response model	*Y*	*χ* ^2^	Conclusion
MOUSE‐INF	Best fitting	Exponential	1.4166	3.842	Best‐fitting model
		Approx. beta‐Poisson	1.4168		—
	Goodness of fit	Exponential	1.4166	7.815	Good fit achieved
		Approx. beta‐Poisson	1.4168	5.992	Good fit achieved
GP‐INF	Best fitting	Exponential	26.50	3.842	—
		Approx. beta‐Poisson	0.725		Best‐fitting model
	Goodness of fit	Exponential	26.50	9.489	Good fit NOT achieved
		Approx. beta‐Poisson	0.725	7.815	Good fit achieved
GERB‐INF	Best fitting	Exponential	5.066	3.842	—
		Approx. beta‐Poisson	1.627		Best‐fitting model
	Goodness of fit	Exponential	5.066	7.815	Good fit achieved
		Approx. beta‐Poisson	1.626	5.992	Good fit achieved

#### 3.1.2 Pooling Analysis of Infection Data

**FIGURE 4:**
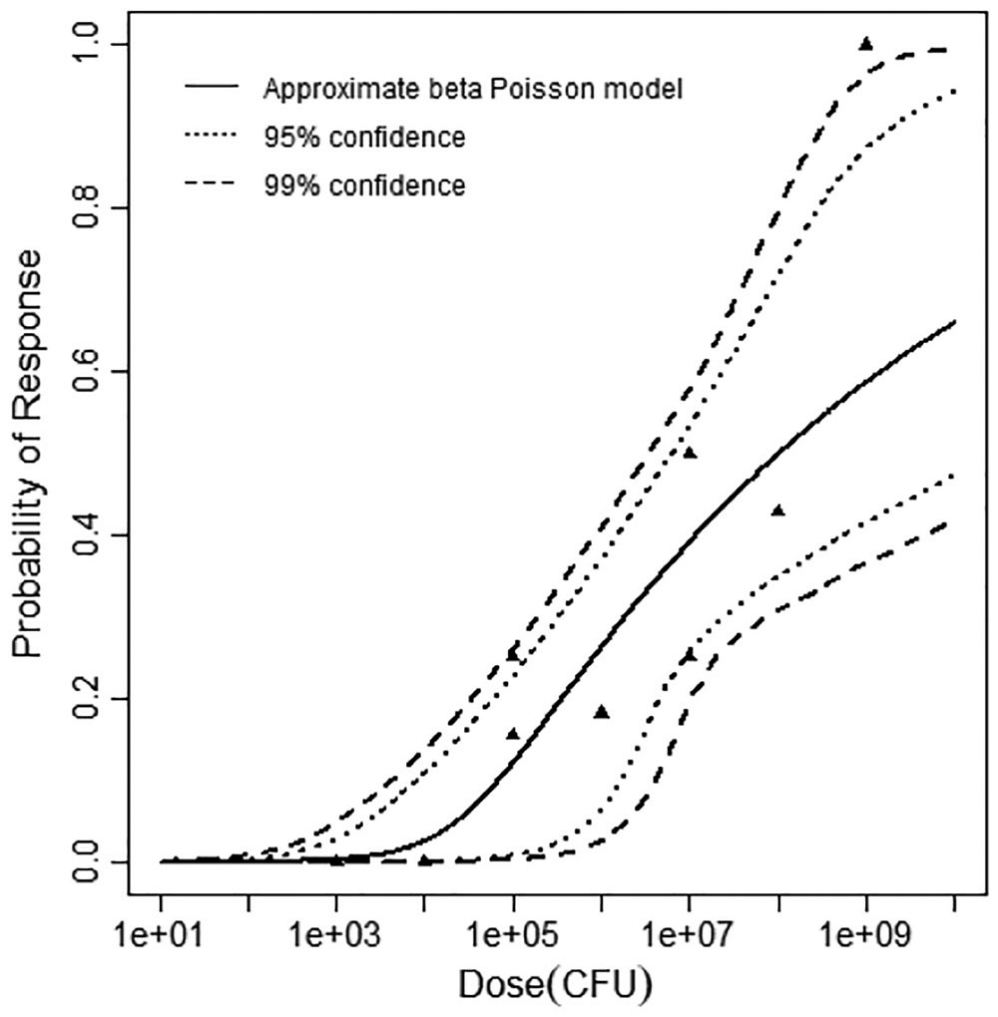
Best‐fitting model (beta‐Poisson) for the pooled guinea pig and gerbil dataset, with 95% and 99% confidence bands.

Since each of the three individual models demonstrated a good fit to at least one of the models, three permutations of nested data sets were combined for pooling evaluations. These three pooled datasets were identified alphabetically with the shared endpoint of infection. The first pooling attempt, labeled A‐INF, included data from mice and guinea pigs, and no good fit to either model was achieved. The second pooling analysis, labeled B‐INF, included the mouse and gerbil animal models; however, no trend was established, and the pooling attempt failed. The guinea pig and gerbil animal model datasets were combined for the third pooling dataset C‐INF, in which the beta‐Poisson model achieved a good fit with the deviance of 6.92 falling beneath the critical chi‐square value of 14.07. A good fit to the exponential model was not achieved. Results of all pooling analyses are summarized in Table 4. The difference in the deviance of the pooled model and the sum of the individual beta‐Poisson model deviances for these data sets was calculated to be 4.569, equal to 6.92 – (0.725 + 1.626). To test whether the data sets could be pooled into a nested model, 4.569 was compared to the critical χ^2^ value at 2 degrees of freedom, 5.991, where the two data sets each had beta‐Poisson models as best‐fitting, and the nested model best fit the beta‐Poisson model (e.g., 4 − 2 = 2). Therefore, the pooling attempt was successful. The pooled model is depicted in Figure 4.

**TABLE 4:** Pooling analysis results for the infection endpoint.

Dataset	Analysis type	Dose‐response model	*Y*	*χ* ^2^	Conclusion
A‐INF	Best fitting	Exponential	130.60	3.842	—
		Approx. beta‐Poisson	17.53		—
	Goodness of fit	Exponential	130.60	15.51	Good fit NOT achieved
		Approx. beta‐Poisson	17.53	14.07	Good fit NOT achieved
B‐INF	Best fitting	*Unable to identify trend*			
C‐INF	Best fitting	Exponential	37.06	3.842	—
		Approx. beta‐Poisson	6.92		Best‐fitting model
	Goodness of fit	Exponential	37.06	15.51	Good fit NOT achieved
		Approx. beta‐Poisson	6.92	14.07	Good fit achieved

#### 3.1.3 Stillbirth Individual Datasets

**FIGURE 5:**
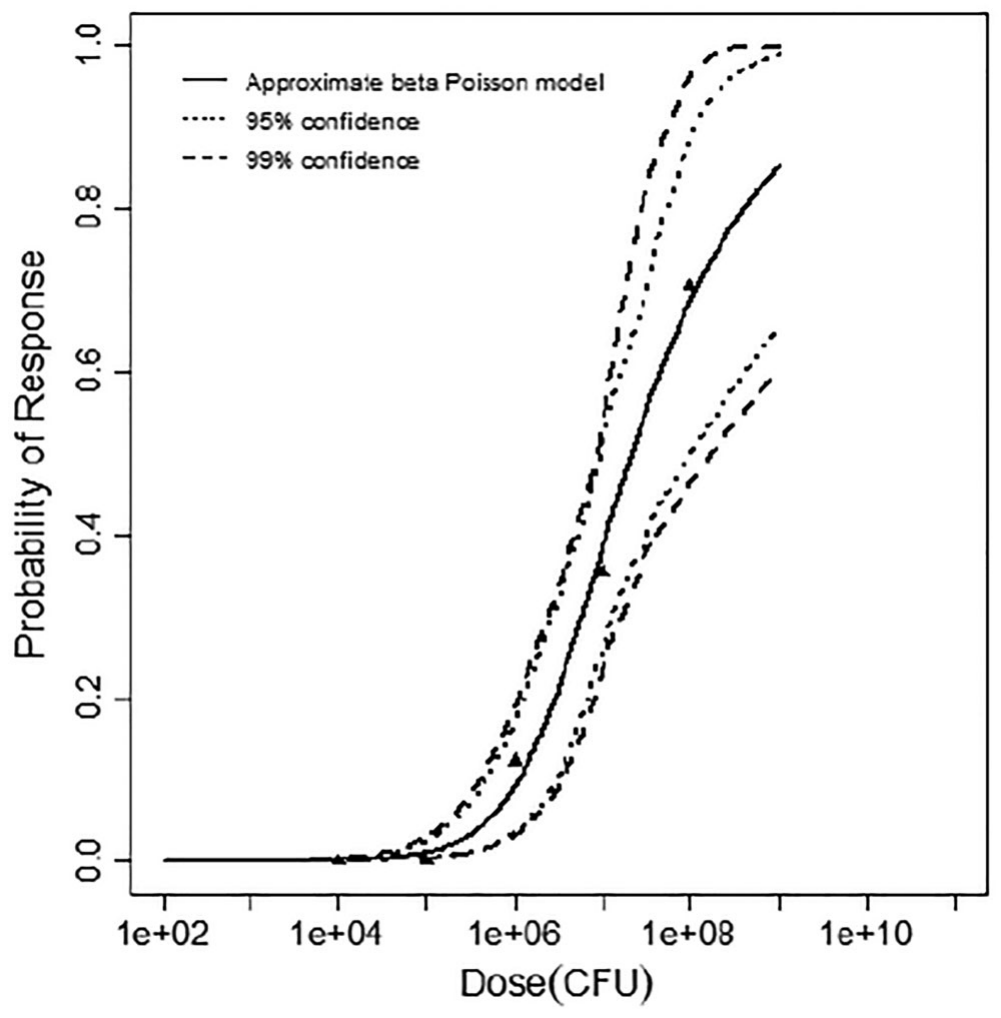
Best‐fitting model (beta‐Poisson) for the guinea pig fetal brain dataset, with 95% and 99% confidence bands.

**FIGURE 6:**
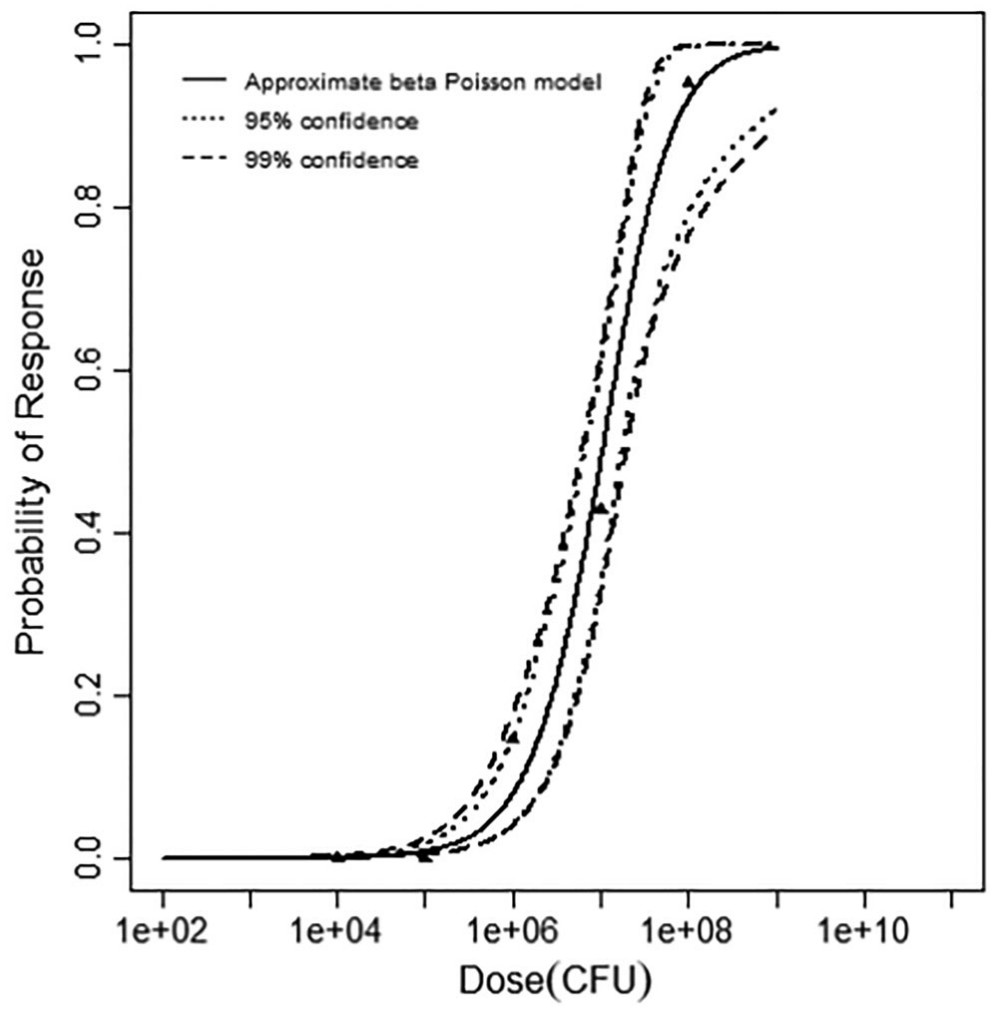
Best‐fitting model (beta‐Poisson) for the guinea pig stillbirth dataset, with 95% and 99% confidence bands.

**FIGURE 7:**
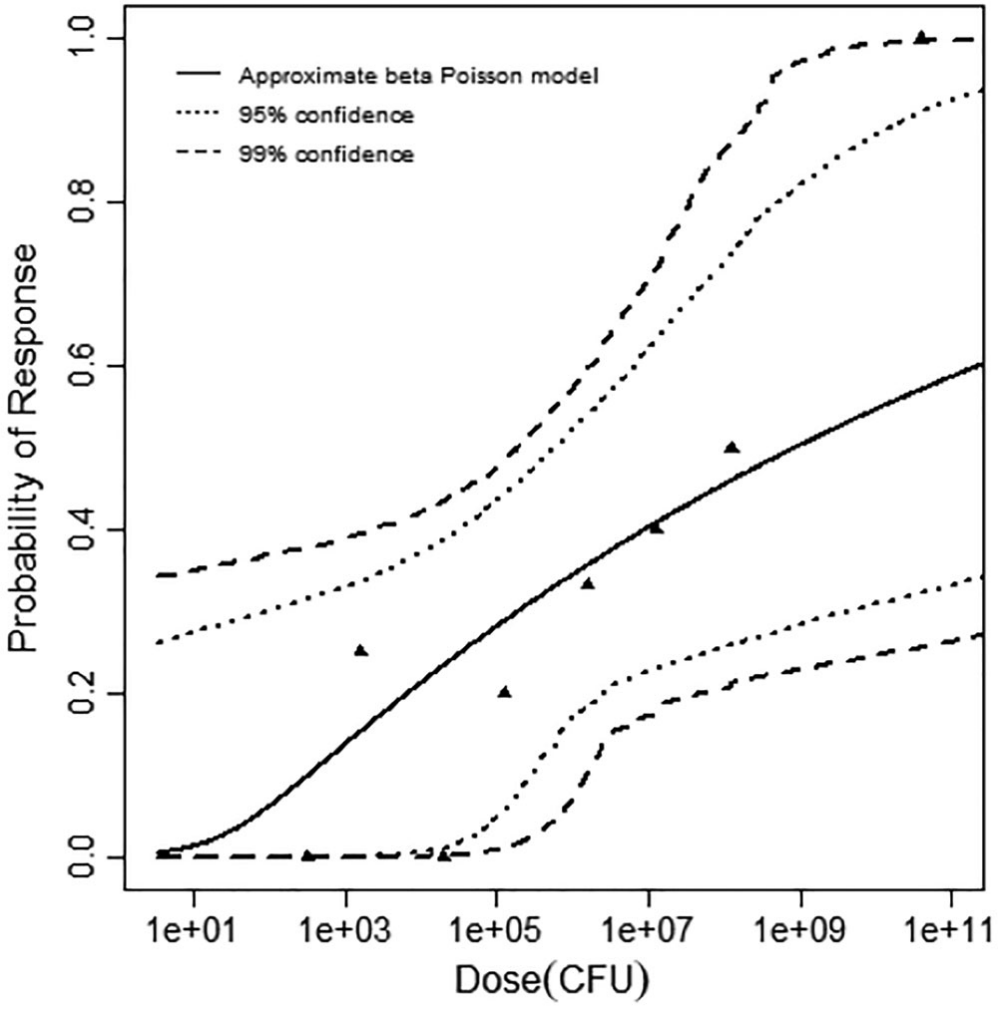
Best‐fitting model (beta‐Poisson) for the rhesus monkey stillbirth dataset, with 95% and 99% confidence bands.

There were three unique stillbirth datasets all fitting at least one dose‐response model. The beta‐Poisson model was a good fit to the GP‐FB dataset (Williams et al. 2007), with a deviance of 1.96, less than the critical *χ* ^2^ value of 7.81. The exponential model did not fit the data set. For the GP‐STILL dataset (Williams et al. 2007), both exponential and beta‐Poisson dose‐response models achieved good fits, with deviances of 8.65 and 3.84, respectively. The best‐fitting model of the two was the beta‐Poisson model, as the difference in the deviances between the two model fits was much less than the critical *χ* ^2^ value at 1 degree of freedom (3.842). The final dataset, MNK‐STILL, described instances of stillbirth in rhesus monkeys (Smith et al. 2008). As for the fetal brain dataset, only the beta‐Poisson model was a good fit for this dataset. The deviance and critical chi‐squared value compared for this dataset and model were3.68 < 3.845 (Table 5). Figures 5, 6, 7 depict the best‐fitting model curves for each data set. A summary of the model fitting output is contained in Table 5.

**TABLE 5:** Stillbirth individual dataset model fitting results.

Dataset	Analysis type	Dose‐response model	*Y*	*χ* ^2^	Conclusion
GP‐FB	Best fitting	Exponential	17.99	3.842	—
		Approx. beta‐Poisson	1.96		Best‐fitting model
	Goodness of fit	Exponential	17.99	9.49	Good fit NOT achieved
		Approx. beta‐Poisson	1.96	7.81	Good fit achieved
GP‐STILL	Best fitting	Exponential	8.65	3.842	—
		Approx. beta‐Poisson	3.84		Best‐fitting model
	Goodness of fit	Exponential	8.65	9.49	Good fit achieved
		Approx. beta‐Poisson	3.84	7.81	Good fit achieved
MNK‐STILL	Best fitting	Exponential	51.36	3.842	—
		Approx. beta‐Poisson	3.68		Best‐fitting model
	Goodness of fit	Exponential	51.36	14.06	Good fit NOT achieved
		Approx. beta‐Poisson	3.68	12.59	Good fit achieved

#### 3.1.4 Pooling Analysis of Stillbirth Data

**FIGURE 8:**
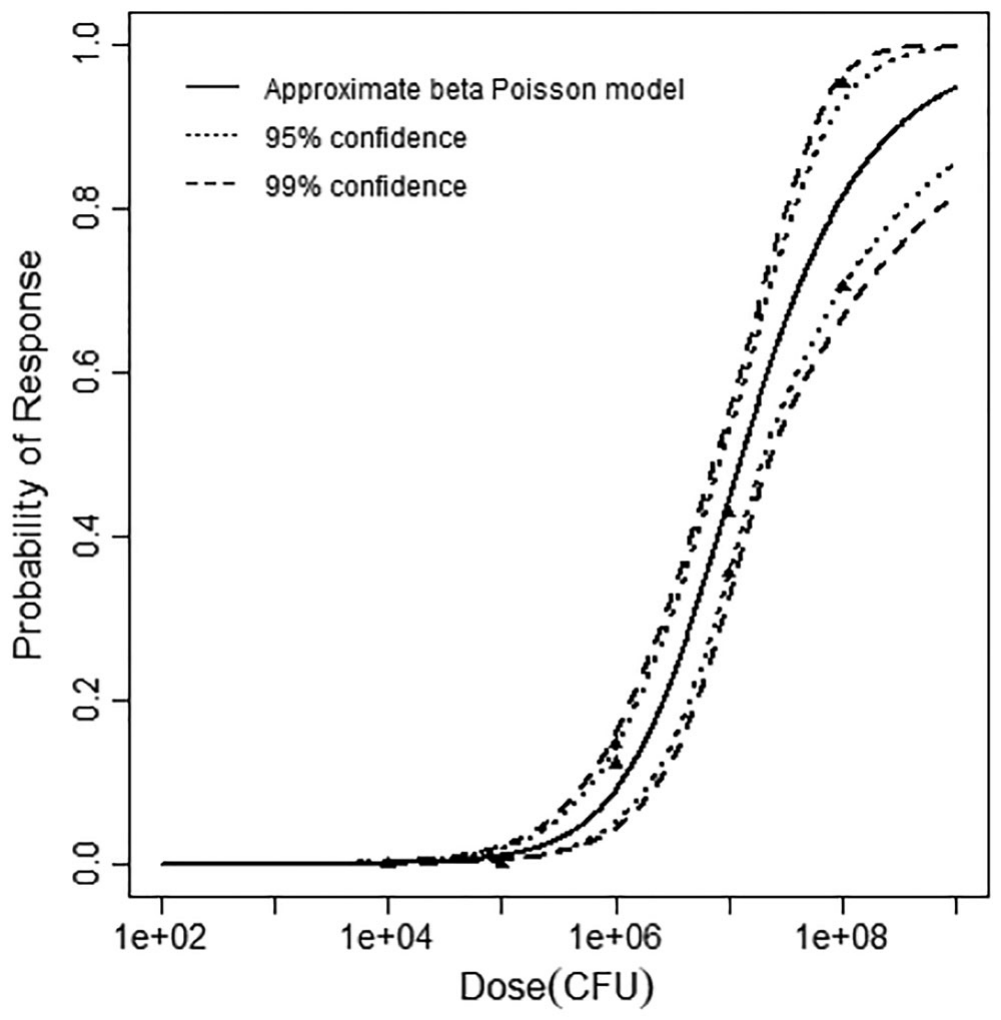
Best‐fitting model (beta‐Poisson) for the pooled guinea pig stillbirth dataset, with 95% and 99% confidence bands.

Three pooling analyses were conducted for datasets with stillbirth as the endpoint. These datasets are labeled as A‐STILL (*GP‐FB* and *GP‐STILL*), B‐STILL (*GP‐FB* and *MNK‐STILL*), and C‐STILL (*GP‐STILL* and MNK‐*STILL*). The only pooling analysis that yielded a good fit was A‐STILL, representing the fetal brain and stillbirth endpoints combined for the guinea pig host. The best‐fitting model for this analysis was the beta‐Poisson model, with a deviance of 10.24 and critical *χ* ^2^ value of and15.51 (Table 6). The difference between the deviance of the nested model (10.24) and the sum of the deviance of the individual beta‐Poisson models (5.8) is 4.44, which is less than the critical *χ* ^2^ value at 2 degrees of freedom, 5.991, indicating a successful pooling analysis. Figure 8 illustrates the dose‐response model for the pooled dataset.

**TABLE 6:** Pooling analysis results for the stillbirth endpoint.

Dataset	Analysis type	Dose‐response model	*Y*	*χ* ^2^	Conclusion
A‐STILL	Best fitting	Exponential	34.64	3.842	—
		Beta‐Poisson	10.24		Best‐fitting model
	Goodness of fit	Exponential	34.64	16.92	Good fit NOT achieved
		Beta‐Poisson	10.24	15.51	Good fit achieved
B‐STILL	Best fitting	Exponential	69.34	3.842	—
		Beta‐Poisson	29.92		—
	Goodness of fit	Exponential	69.34	21.03	Good fit NOT achieved
		Beta‐Poisson	29.92	19.68	Good fit NOT achieved
C‐STILL	Best fitting	Exponential	64.43	3.842	—
		Beta‐Poisson	36.95		—
	Goodness of fit	Exponential	64.43	21.03	Good fit NOT achieved
		Beta‐Poisson	36.95	19.68	Good fit NOT achieved

### 3.2 Evaluation for Final Model Recommendations

#### 3.2.1 Recommended Dose‐Response Model for Infectious Endpoint

A summary of the parameters for all datasets that produced an acceptable fit is included in Table 7. Since Δ = 4.569 is less than 5.991 the critical *χ* ^2^ value at 2 degrees of freedom, for the pooled guinea pig and gerbil infection datasets (C‐INF), interspecies nesting for the infection endpoint was successful. This indicates that the data sets come from a similar distribution. Therefore, inferences that the variation in dose‐response between guinea pigs and gerbils are relatively minor. The nested model provides a more robust dose‐response curve for utilization across species and is therefore recommended (Huang et al. 2009; Bartrand et al. 2008, Breuninger and Weir 2015). The parameters of the approximate beta‐Poisson for this model are *α* = 0.0843 and *N_50_* = 1.01 × 10^8^.

**TABLE 7:** Infection model summary and final recommendation.

Dataset	Best‐fitting model	*k*	*α*	N50	Comment
MOUSE—INF	Exponential	3.15 × 10^−3^			—
GP‐INF	Approx. beta‐Poisson		4.95 × 10^−2^	1.12 × 10^10^	
GER‐INF	Approx. beta‐Poisson		2.32 × 10^−1^	1.21 × 10^6^	—
C‐INF (*GP and GERB*)	Approx. beta‐Poisson		8.43 × 10^−2^	1.01 × 10^8^	Recommended model

#### 3.2.2 Recommended Dose‐Response Model for Stillbirth Endpoint

Table 8 provides a summary of the model parameters from the analysis for the stillbirth endpoint. Based on the results of the pooling analysis, a nested model was produced with the combined guinea pig data sets. Despite differences in the endpoints, the data could be fit to a model with the same functional form, indicating that they come from a common distribution and allowing for inferences that the variation between these endpoints is relatively small. Combined, the A‐STILL model is more robust than any of the individual models, reducing uncertainty; therefore, it is the recommended dose‐response function for consideration in quantitative microbial risk assessments.

**TABLE 8:** Final stillbirth recommended model evaluation.

Dataset	Best‐fitting model	*α*	N50	Comment
GP‐FB	Approx. beta‐Poisson	3.27 × 10^−1^	2.15 × 10^7^	
GP‐STILL	Approx. beta‐Poisson	1.38	1.05 × 10^7^	—
MNK‐STILL	Approx. beta‐Poisson	4.00 × 10^−2^	8.22 × 10^8^	—
A‐STILL (*GP‐FB and GP‐STILL*)	Approx. beta‐Poisson	0.575	1.31 × 10^7^	Recommended model

A‐STILL follows the approximate form of the beta‐Poisson model, with parameters of *α* = 0.575and *N_50_* = 1.31 × 10^7^.

#### 3.2.3 Summary and Recommendation

Ultimately, this study provides recommended models for two dose‐response endpoints (infection and stillbirth) for use in risk assessments for oral exposure of *L. monocytogenes* in pregnant individuals. There is no single model that best represents all pathogen‐ and exposure route‐specific dose‐response relationships. Rather, for each QMRA, it is vital to ensure that the dose‐response model accurately reflects the scenario under consideration and is health protective for the target population. To compare the models developed herein to previously published models, the *N_50_*, which represents the pathogen dose resulting in 50% of exposed subjects experiencing the adverse health outcome (infection or stillbirth), was used. We note that the *N_50_* represents just one point or region in the curve, and when selecting a dose‐response model, a comparison should be made within the exposure dose region most applicable. The final recommended models have some of the lowest *N_50_* values of all those generated in this analysis and thus provide the most conservative risk estimates. Following this method, the two recommended models for infection and stillbirth from the present study were compared with models currently available in the literature to corroborate the utility of sensitive population‐specific dose‐response models in public health decisions.

## 4 Discussion

### 4.1 Comparative Evaluation With Published Dose‐Response Models

**FIGURE 9:**
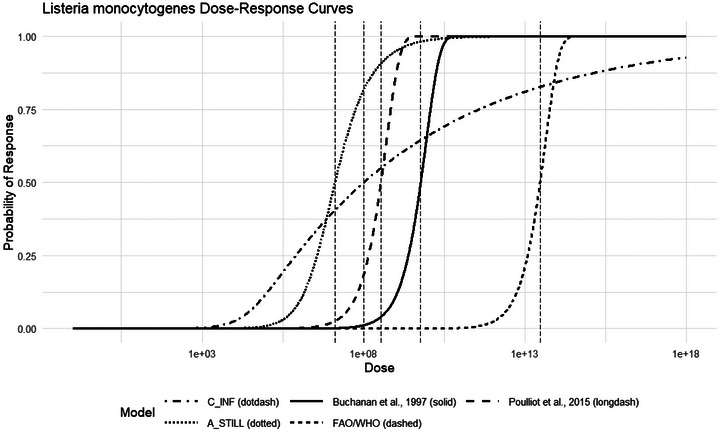
Comparison of dose‐response models developed herein with previously published models. *N_50_* values for each model are depicted by vertical dashed lines.

There is a previously published existing dose‐response model specific to *L. monocytogenes¸* oral exposure, and sensitive populations derived from epidemiological data and used for the infection endpoint (Buchanan et al. 1997). Herein, three novel dose‐response models were developed using the datasets from pregnant animal studies in mice (Golnazarian et al. 1989), Guinea pigs (Williams et al. 2007), and gerbils (Roulo et al. 2014). Collectively, these models represent available dose‐response relationships of orally ingested *L. monocytogenes* specific to infection outcomes, providing adequate means to compare across models to draw claims about recommendations for risk assessors. While derived using surveillance data, which is most representative of realistic exposures, such data is more uncertain than animal challenge studies with controlled dosing. The *N_50_* of the Buchanan et al. model is approximately 5.25 E9. The recommended infection dose‐response model herein was the approximate beta‐Poisson model fit to pooled data from guinea pigs and gerbils, with an *N_50_* value of 1.01 × 10^8^. While these values are similar and the Buchanan et al. *N_50_* is within the confidence region of the current recommended model, it should be noted that the previous model, which was purposefully derived to be conservative, may provide a less health protective estimate for oral exposure to *L. monocytogenes* in pregnancy, as it is for the illness endpoint (symptomatic listeriosis) rather than infection. Also developed using epidemiological data Pouillot et al. (2015) created a dose‐response model for sensitive, highly susceptible subpopulations, including pregnant women, among several other health conditions, and reported an *N_50_* of 3.49 × 10^8^, which is very close to the *N_50_* of the infection model herein. Similarly, FAO/WHO 2004 provided a model for more susceptible subpopulations not specific to pregnant individuals with an *N_50_* of 2.93 E13. Figure 9 compares both the recommended models developed herein for infection and stillbirth in pregnant hosts with the three previously published models for immunocompromised conditions in general. It should be noted that the Pouillot et al. (2015) model is based on the reported mean of the exponential model parameter, which may be more sensitive to outliers than the median. Overall, the infection model produced herein falls within the range of previously published models.

The stillbirth model comparison differs slightly. There is one dose‐response model specific to *L. monocytogenes*, oral exposure, pregnant individuals, and an endpoint of stillbirth or equivalent for comparison. The Williams et al. study was one in a series of studies investigating oral ingestion of *L. monocytogenes* in pregnant guinea pig and rhesus monkey animal models, reporting two separate logistic dose‐response models for each animal model (Williams et al. 2009, Erratum 2011). However, this study did not fit data to the beta‐Poisson model, and was not pooled, as in the present work. LD50 values for the Williams et al.’s guinea pig and rhesus monkey data fitting analyses were 2 × 10^7^ and 4 × 10^7^, respectively (Williams et al. 2009). The study herein recommends the pooled guinea pig model with an LD50 of 1.31 × 10^7^, which is comparable to the Williams et al. values. Guinea pig fetal brain data were not considered in the Williams et al. (2009) models.

### 4.2 Limitations

There are several limitations in this work. First, as with any microbial dose‐response model, outcomes at low doses are difficult to predict; as such, the data from feeding studies utilized for model development were tested at higher doses (minimum 10^2^–10^4^) (Smith et al. 2008; Williams et al. 2007). Second, the datasets used to build the current model were from animal studies. Animal dose‐response models may diverge from human dose‐response by genetic variability, longevity, and exposures to microbial and chemical health hazards (Crump et al. 2010). However, the animal feeding studies considered in the present work were carefully selected to ensure equivalent disease mechanisms and progression (e.g., gastroenteritis and placental transmigration) and are regarded as the most appropriate feeding studies available. In addition, when compared to all existing animal studies, the animal models chosen, guinea pigs and non‐human primates, have the most similar placental anatomies to humans (Lowe et al. 2018). Specifically, guinea pigs are preferred for modeling human fetal effects for their parallel placental structures, numerous fetuses per litter, and economical benefits (D'Orazio 2014). Despite this, there are still research gaps that future listeriosis animal studies may address. Because *L. monocytogenes* is readily destroyed in the human stomach, it is imperative to choose an animal model with analogous stomach pH and gastric bile characteristics; unfortunately, these prospective disparities have not yet been explored (D'Orazio 2014). In addition, guinea pig InlB may not interact with Met receptors in a similar capacity as in humans, although it is not fully understood how this may affect placental invasion (Lowe et al. 2018). Lastly, the mechanism leading to increased stillbirths after early gestational exposure is still unclear (Lowe et al. 2018). Investigating these pathogenesis characteristics could improve dose‐response modeling for bacterial infections causing stillbirth in the future.

### 4.3 Implications and Considerations

The dose‐response model evaluations conducted within this study have provided several insights. The initial development of the dose‐response model took into consideration the stillbirth endpoint as it was reported within the current literature. One point of convolution pertaining to the present dose‐response modeling is the time point from the initial infection and the target endpoint for listeriosis in pregnant hosts. To address this, we included datasets representative of two distinct endpoints: infection and stillbirth. Given that stillbirth results from invasive infection, this two‐endpoint approach supports examining varying disease contexts.

The recommended stillbirth model employing fetal brain infection data is another result warranting elaboration. The incorporation of the guinea pig fetal brain infection data was grounded in the aforementioned consistent cooccurrence of stillbirth and fetal brain infection. Beyond this, stillbirth is a difficult endpoint to recover from host subjects, as in the studies included, animals were often sacrificed before stillbirth could naturally occur (Smith et al. 2008; Williams et al. 2007). Fetal brain infection is a verifiable, recoverable endpoint in sacrificed animals that is nearest to stillbirth. As such, both endpoints aligned with current theoretical underpinnings for the use of animal models in dose‐response modeling (Haas et al. 2014). The results of the pooling analysis must also be considered (D'Orazio 2014). Pooling is typically possible with datasets representing similar mechanistic endpoints. Therefore, the successful pooling of the fetal brain infection data with other stillbirth datasets provides evidence for the recommendation of a stillbirth dose‐response model based on fetal brain infection data. Overall, the use of multiple endpoints and animal models is believed to have only strengthened this analysis.

## 5 Conclusion

The approximate beta‐Poisson dose‐response models developed in this analysis were the best‐fitting, existing biologically plausible dose‐response models available for estimating risks to pregnant people orally exposed to *L. monocytogenes*. While the exact beta‐Poisson model form was also explored, it did not provide significantly different parameter estimates or deviance values than the approximate parametrization of the model, as expected given the range of parameter values in this study. It was not included in this work for this reason. The novel employment of fetal brain infection data for stillbirth modeling produced a better‐fitting model than those previously developed and highlighted the utility of a more observable surrogate endpoint. The *N_50_* values from the current models are similar to, though more conservative than, previous models, thereby providing an option for more health protective risk estimation. While limiting potential overestimation of risk is an important consideration for the risk assessor when applying these models, scaling to epidemiological data that are not specific to the immunocompromised condition or the health endpoint of interest should be undertaken cautiously. Pregnancy is confounded with multiple physiological, behavioral, and clinical variables, and therefore should not be treated simply as a general immunocompromised state. The increase in foodborne listeriosis outbreaks continues to challenge food safety policy‐making. Therefore, the development of dose‐response models specific to endpoints faced by sensitive populations provides an opportunity for risk assessors and public health decision makers to better characterize probabilities of adverse outcomes, ultimately enhancing health‐protective strategies.
